# First Record of *Hesperobaenus rufipes* (Coleoptera: Monotomidae) in South America

**DOI:** 10.1007/s13744-026-01448-w

**Published:** 2026-10-28

**Authors:** Flávia Fagundes de Paula, Ana Claudia da Silva, Jamilly Raniely Batista Barbosa, Leonardo Peres Sartori, Raul Alberto Laumann, Juares Fuhrmann, Eliana Maria Gouveia Fontes

**Affiliations:** 1 Instituto Federal Goiano, Rio Verde, GO Brazil; 2 Univ Federal de Goiás, Goiânia, GO Brazil; 3 Empresa Brasileira de Pesquisa Agropecuária, EMBRAPA Recursos Genéticos E Biotecnologia, Brasília, Federal District Brazil; 4 Instituto Nacional de Coleoptera (INCol), Cuiabá, MT and Museu de Zoologia da Univ de São Paulo, São Paulo, SP Brazil

**Keywords:** Brazil, Cerrado, Corn, Invasive, Soybean

## Abstract

This study reports the first record of *Hesperobaenus rufipes* LeConte, 1863 (Coleoptera: Monotomidae) in South America, based on specimens collected in Brazil, specifically in the state of Goiás. The beetles were sampled from the soil in soybean and corn fields using pitfall traps. This represents the first record of the species beyond its native range in North America, and its introduction pathway remains unknown.

The family Monotomidae comprises 36 genera and 257 described species worldwide (McElrath and McHugh 2018). Although a small fraction of these taxa exhibits a cosmopolitan distribution, most species are characterized by restricted geographical ranges (McElrath and McHugh 2018). Commonly known as “minute clubbed beetles,” they are small (1.5–6.0 mm) and inconspicuously colored, with detritivorous, mycophagous, or predatory habits (Grégoire et al. 1985; McElrath and McHugh 2018).

Representing this diversity, *Hesperobaenus rufipes* LeConte, 1863 (Coleoptera: Monotomidae) is a species native to North America, widespread in the eastern USA (McElrath and McHugh 2018). In its native range, this beetle inhabits the subcortical environment of hardwood trees, such as *Acer*, *Carya*, *Fagus*, and *Quercus*, where its diet consists primarily of fungal hyphae (Justis 2009; McElrath and McHugh 2018).

Despite its documented presence in the Nearctic region, there are no prior records of its occurrence or establishment in other biogeographical zones. This record documents the presence of *H*. *rufipes* in South America for the first time, specifically within the Cerrado biome in Brazil, representing a significant expansion of its known geographical range.

This observation resulted from broader field surveys conducted on soybean and corn fields as part of the Regenera Cerrado Project, in which the soil entomofauna was sampled across nine farms (1339.8 ha) located in the municipalities of Montividiu and Rio Verde, Goiás State, Brazil. The Regenera Cerrado Project is an initiative that brings together farmers, researchers, and technical support networks to evaluate, validate, and promote the adoption of regenerative agriculture practices in grain production systems in the Brazilian Cerrado. *Hesperobaenus rufipes* was detected in seven farms. The farms are located at the following coordinates: site 1 (17.7381° S, 51.2881° W), site 2 (17.4812° S, 51.4603° W), site 3 (17.3476° S, 51.3822° W), site 4 (17.4200° S, 51.3471° W), site 5 (17.3976° S, 51.4069° W), site 6 (17.6534° S, 51.0860° W), and site 7 (17.4560° S, 51.4695° W).

In November 2023, at the onset of the rainy season in the Cerrado biome, 24 adult specimens were collected from soybean fields using unbaited pitfall traps. An additional adult was collected from a corn field in April 2024, during the transition from the rainy to the dry season. Each field was divided into four quadrats, with one pitfall trap installed in each quadrat, resulting in four traps per field. Traps were positioned at least 50 m from field margins to minimize potential edge effects.

Each pitfall trap consisted of a glass vial (8.5 cm high × 3.5 cm in diameter) containing 50 mL of 70% ethanol as a preservative and placed inside a larger plastic container (15 cm high × 9.5 cm in diameter). The plastic containers were buried to a depth of 15 cm, and a polyethylene funnel was positioned flush with the soil surface above each container to direct captured arthropods into the vial. Traps were deployed once during the soybean flowering stage and once during the corn tasseling stage and remained active for 48 h. Taxonomic identification was performed using the identification key provided by Bousquet (2002). The specimens were deposited in the Entomological Collection of the Museu de Zoologia da Universidade de São Paulo (MZUSP) and dry-mounted on card triangles using water-based, acid-free glue. Material examined (15): Brazil, Goiás, Montividiu, soybean crop, “Fazenda Pai Noel,” − 17.7380667, − 51.28812, 09.ix.2023, pitfall trap, Projeto Regenera Cerrado (collectors), 2 specimens (MZUSP 74930–74931); “Fazenda Bom Jardim,” − 17.4199535, − 51.3471454, 14.ix.2023, pitfall trap, Projeto Regenera Cerradao (collectors), 3 specimens (MZUSP 74932–74934); “Fazenda Segredo,” − 17.48124, − 51.460295, 27/09/2023, pitfall trap, Projeto Regenera Cerradao (collectors), 1 specimen (MZUSP 74935); “Fazenda Brasilandia,” − 17653417, − 51,086017, 27.ix.2023, pitfall trap, Projeto Regenera Cerradao (collectors), 9 specimens (MZUSP 74936–74944).

The collected adults of *H. rufipes* have body (Fig. 1A–C) length 2.0–2.8 mm and dorsal surface dark reddish-brown, some specimens with antennae, elytra, and legs slightly light. The head (Fig. 1D, E) exhibits distinct sexual dimorphism, being wider in males than in females. In both sexes, the antennae present the antennomere IX wider than long, which is noticeably narrower than antennomere X (Fig. 1D, E). The pronotum is transverse, reaching its maximum width just before the apex, with rounded and non-produced anterior angles (Fig. 1A). Its disk is slightly convex (Fig. 1B), featuring a relatively wide median impunctate longitudinal area, while the lateral punctures are closely spaced but not confluent. The elytra are elongated, bearing an oblique depression on the anterior third near the suture, and the strial punctures are coarse and deeply impressed, four striae are distinct at elytral lateral inflection (Fig. 1B). The scutellum is devoid of setigerous punctures. Ventrally, the prosternal apophysis lacks microsculpture. The first visible abdominal sternite has a triangularly produced coxal bead (Fig. 1C), which may occasionally exhibit a short longitudinal extension. The last abdominal visible tergite exposed dorsally when elytra in rest position (Fig. 1F).

**Fig. 1:**
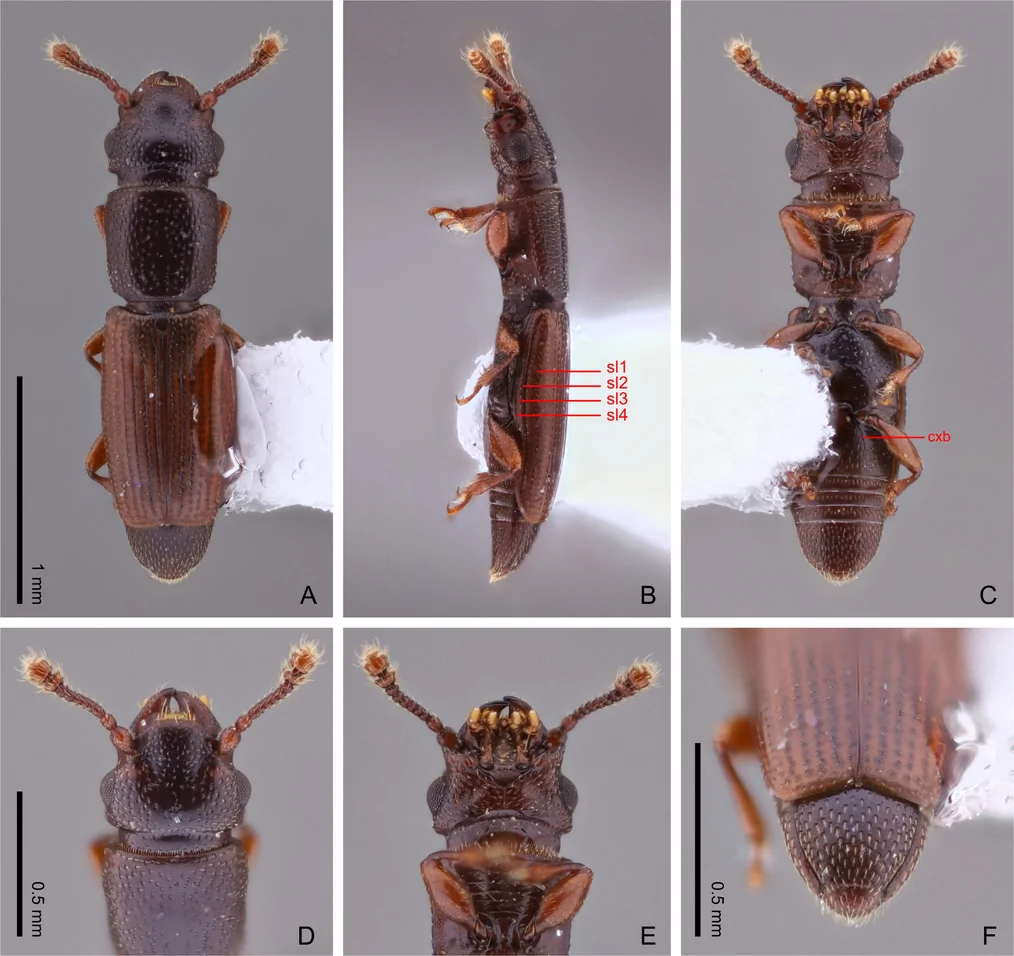
*Hesperobaeus rufipes*, habitus dorsal (**A**), lateral (**B**), and ventral (**C**); head dorsal (**D**), ventral (**E**); dorsal view of posterior area of abdomen (**F**). Specimen collected from the state of Goiás, Brazil. cxb, coxal bead of first abdominal visible sternite; sl1–4, elytral lateral striae 1–4

This record highlights a significant gap in the known distribution of *H. rufipes* and registers its possible establishment in a novel ecological context. Although characterized as a mycophagous species usually associated with the subcortical environment of hardwood trees, its occurrence in the soil of soybean and corn fields suggests a potential niche expansion within Neotropical agricultural systems. Furthermore, while *H. rufipes* is a non-native species in this region, its introduction pathway remains unknown.
